# Association Between Dietary Fluoride and Calcium Intake of School-Age Children With Symptoms of Dental and Skeletal Fluorosis in Halaba, Southern Ethiopia

**DOI:** 10.3389/froh.2022.853719

**Published:** 2022-03-04

**Authors:** Nahom Tefera, Demmelash Mulualem, Kaleab Baye, Masresha Tessema, Meseret Woldeyohannes, Asrat Yehualashet, Susan J. Whiting

**Affiliations:** ^1^Ethiopia and Food Science and Nutrition Research Directorate Ethiopian Public Health Institute, Center for Food Science and Nutrition, College of Natural and Computational Sciences Addis Ababa University, Addis Ababa, Ethiopia; ^2^School of Human Nutrition and Food Science, Hawassa University, Awasa, Ethiopia; ^3^Center for Food Science and Nutrition, College of Natural and Computational Sciences Addis Ababa University, Addis Ababa, Ethiopia; ^4^Food Science and Nutrition Research Directorate Ethiopian Public Health Institute, Addis Ababa, Ethiopia; ^5^College of Pharmacy and Nutrition, University of Saskatchewan, Saskatoon, SK, Canada

**Keywords:** fluoride, fluorosis, calcium, dental, skeletal, school-age children, Ethiopia

## Abstract

**Background:**

In the Ethiopian Rift Valley, ways to reduce the fluoride (F) burden from drinking water have been unsuccessful. Calcium (Ca) intake may mitigate fluorosis by binding with F ions and preventing absorption. The purpose of this study was to examine the association between Ca intake and proportion of fluorosis symptoms in school-age children in an area where F levels are known to be higher than WHO limit of 1.5 mg F/L water.

**Methods:**

A cross-sectional survey in the Halaba zone involved 135 eligible children aged 6–13 year who were recruited to have dental fluorosis assessed by a dentist and skeletal fluorosis assessed by a physiotherapist. Dietary Ca intake was determined by 24-h recall. Food items and samples from ground wells, taps and spring water were collected for F concentration. Associations were measured using bivariate logistic regression, adjusted for known confounders.

**Results:**

Water F averaged 5.09 mg/L. Total F intake was high, 10.57 mg/day, and Ca intake was low, 520 mg/day. Prevalence of dental fluorosis (from very mild to severe symptoms) was 73.1% for younger children (6–8 years) and 68.3 % for older children (9–13 years). The prevalence of children having symptoms of skeletal fluorosis ranged between 55.1 and 72.4%, with no apparent age difference. Dietary F intake of children was significantly positively associated with presence of dental fluorosis. Dietary Ca intake of children was significantly negatively associated with dental fluorosis. Higher than average dietary F intake significantly increased the odds of developing skeletal fluorosis symptoms when measured as inability to stretch and fold arms to touch back of head. Higher than average Ca intake was significantly associated with decreased odds of developing skeletal fluorosis measured as inability to bend body to touch the toes or floor.

**Conclusions:**

High dietary F, as expected, was associated with fluorosis in children. In the presence of higher Ca intake (>520 mg/day) some fluorosis symptoms were mitigated. There is a need to improve Ca intakes as all were below recommended levels, and this nutritional strategy may also reduce burden of excess F.

## Background

Fluoride (F) is a micronutrient having both an Adequate Intake (AI) recommended intake and a Tolerable Upper-Intake Level (UL) from the Institute of Medicine [1]. Its role is primarily to form fluorapatite, which makes bone stronger and teeth more resistant to decay. Prolonged ingestion of F at levels above the UL leads to mottled then damaged teeth (dental fluorosis) and deformed bones (skeletal fluorosis). Non-skeletal conditions such as thyroid problems, growth retardation, kidney damage, can arise [2]. Children are vulnerable to dental fluorosis as baby teeth and subsequently, permanent teeth, erupt during childhood. The AI of F for children ages 4–8 and 9–13 years is 1 and 2 mg/day, respectively. The ULs for these age groups are 2.2 and 10.0 mg/day, respectively, showing a very narrow safe range of intake [1].

The major source of ingested F is in drinking water [3, 4], and the WHO [5] has set limits on drinking water F concentration of 1.5 mg/L. Millions of people worldwide rely on drinking water with F concentrations exceeding this level, including those in the Great Rift Valley that extends through eastern Africa [6]. In the Ethiopian Rift Valley, the average F content of ground water used for drinking is 6.03 mg/L and as much as 28% of the population has dental fluorosis [7]. Afar, Oromia, and SNNPR are the Ethiopian regional states which are most affected by high F level in the drinking water supply [8], and some areas are greatly affected; for example, the prevalence of dental fluorosis in rural communities of the Ziway-Shala lake basin was reported as 62% [9].

The severity of fluorosis is associated not only with F dose, but also with other factors such as length of exposure, altitude, and nutritional status [10, 11]. Fluoride is efficiently absorbed from both the stomach and small intestine without regulation [12]. However, studies in Ethiopia showed that people in diverse regions exposed to similar water F levels had different degrees of fluorosis severity in children [13], and variations in dietary calcium (Ca) intakes were implicated. Calcium, a cation, binds to F, an anion, to form insoluble salts that are not absorbed. A study performed using an animal model reported that calcium-magnesium salts or a plant source of these minerals effectively reduced apparent F absorption, indicating support for a calcium-induced reduction in fluorosis development [14].

In Ethiopia, the extent of dental and skeletal fluorosis has been assessed in children in communities having different calcium intakes [14, 15]; however, an association of dietary calcium intake with fluorosis using individual data has not been demonstrated. This study aimed to assess whether the prevalence of dental and skeletal fluorosis was associated with calcium intake in school-age children ages 6–13 years in Halaba, Southern Ethiopia, an area known for high F water levels [13].

## Materials and Methods

This study was carried out in Halaba zone, the southern part of Ethiopia. It is situated on an average altitude of 1,800 m above sea level with an average 750 mm annual rainfall which is classified as dry midland; it is located in the Ethiopian Rift Valley. The study area was selected based on a past report showing a high-water F content in the community (~5 mg/L) [13].

### Study Design

The study design was a cross-sectional survey in the village (kebele) of Lay Arsho in the Halaba zone. In the kebele, households had been identified for a larger study [16] and those with eligible school-age children were invited to participate in this sub-study.

### Study Period

Dental and skeletal fluorosis assessments were done in January 2018 during a larger survey involving two kebeles [16] during which time the same study personnel also collected data from the mothers of these school-age children, including dietary calcium using a food frequency questionnaire. The dietary intake data by 24-h recall, food, and water samples were collected in January 2019. Fluoride levels of food and water samples were analyzed in April-May 2019.

### Target and Study Population

The study population was all eligible school-age (6–13 years) children living in the study area for at least 6 months prior to this study. In this age group, dental fluorosis can be identified, and prevalence rates can be compared to previous studies. Those children who reported having any illness in January 2018 were excluded from the study. The sample size was estimated using EpiInfo Version 7.0.8.3 by considering 12% prevalence of skeletal fluorosis among school-age children in the same study area [13], 95% level of confidence and absolute precision (*d* = 5%). Since the total study population was <10,000, a finite population correction formula was used to correct the calculated sample size. Finally, based on the above assumptions and by considering a 10% non-response rate, a minimum sample size of 135 school-age children was estimated as sufficient power to determine the level of fluorosis among school-age children.

### Dental and Skeletal Fluorosis Assessment

School-age children were assessed for the presence of dental fluorosis based on the Dean index [17] which classifies dental fluorosis into 6 categories: normal, questionable, very mild, mild, moderate, and severe dental fluorosis. Further details have been published elsewhere [13]. All examinations were conducted at a health post. The examination of teeth was carried out by a qualified, experienced Ethiopian dentist. Subjects were instructed to come having thoroughly brushed their teeth. Skeletal fluorosis rates among school-age children were assessed by a qualified, experienced Ethiopian physiotherapist using published clinical symptoms and physical exercises specifically for this purpose [13, 18, 19]. Individuals who could not perform the physical exercise in the endemic areas, as well as display stiffness of the back and neck muscles, unable to bend forward or to stand straight were categorized as having skeletal fluorosis [13].

### Determination of Fluoride From Food and Water

During the dietary intake data collection in 2019, 20 types of food items consumed by the households were identified. From each category of identified food items, at least 0.5 kg of each food sample was collected from households by the composite sampling method. In the study area, the community used tap, ground, and spring water sources, therefore a one-liter water sample was collected from each of these water sources.

Food samples collected from different households were dried, ground, and homogenized using a drying oven, miller, and homogenizer, respectively, for F analysis. Samples of 0.5 g were weighed to the nearest 0.1 mg directly into nickel crucibles. The samples were covered with 5.0 ml of 8 M NaOH, and carefully mixed. The crucibles were put on a hot plate for evaporation to dryness before they were covered and put into the muffle furnace for combustion. The temperature program for the muffle furnace was set at 200°C for ~16 h after which the temperature was increased to 525°C and kept there for 3 h. The crucibles were cooled, 15 ml distilled water was added, and the crucibles were put on a hot plate to aid the dissolution of the fusion cake. After ~2 h, the sample solutions were transferred to 50 ml capped plastic tubes. The sample solutions were neutralized using concentrated and then diluted HCl. Concentrated HCl was added dropwise until the pH decreased from 12.0–13.0 to 8.0–8.5. Diluted HCl was added dropwise until the pH decreased from 8.0–8.5 to 7.2–7.5. The pH meter was calibrated by pH buffers 7.0 and 10.0. The sample solution was diluted to 50 ml with distilled water and stored in 50 ml air-tight plastic tubes until analyzed. Aliquots of 5 ml were taken out for analysis after the ash had settled and the solutions were clear. Care was taken to avoid the settled ash. Before analyzing, 0.5 ml TISABIII was added to obtain a pH of 5.2–5.4, which is the optimal pH-range for F determination. Reagent blanks were always prepared together with the samples. The F levels of standard solutions and sample solutions were measured in ppm using a fluoride ion-selective electrode. For all food samples, 1 ml of 100 ppm F standard was spiked for the verification of the accuracy of the method in terms of % recovery [20].

For community water analysis, 5 ml of buffer solution was added followed by 5 ml of the water sample, into a measuring cell. An equal amount of buffer solution was also added for working standards. The electrode was calibrated by working standards starting from most diluted to concentrated standard. The concentration of the solutions was directly measured in ppm by using a F ion-selective electrode. A detailed description of this method has been published [13].

### Dietary Intakes

The type and amount of food consumed by the study participants were collected by a 24-h dietary recall method using a pre-structured questionnaire developed based on internationally recognized multiple-pass methods [21]. Additionally, the dietary intake data for children was collected by a pre-structured 16 item food frequency questionnaire (FFQ) that focused on foods in the region that were good sources of calcium, following Wu et al. [22] methodology for a calcium FFQ as we described recently [16] for use in Ethiopia. The Ethiopian food composition table was used to estimate the dietary calcium intake of study subjects. Total F intake was estimated by using food and water F content analyzed in this study and from values from our previous research [13].

### Data Analysis

The 24-h dietary data was estimated by Nutri-survey software whereas Microsoft excel was used for FFQ data estimation. Data were entered and analyzed using SPSS version 20. Bivariate logistic regression was used to look for the association between the dependent and independent variables. All variables which show statistical associations at *p* < 0.25 during bivariate analysis were a candidate for the multivariate analysis. For dietary Ca and F, the intake derived from the 24-h recall was used in regression analyses. Multivariate logistic regression analysis was done to see the association between the independent variables with the dependent variable by controlling possible cofounders. Lastly, AORs at 95% a confidence interval (CI) with a *p* < 0.05 were used as statistically significant predictors and reported. Descriptive statistics, such as mean and frequency were used to display study results.

### Ethical Clearance

Ethical approval was obtained from Addis Ababa University (AAU) with the reference number CNSDO/204/11/2018. The study was conducted according to rules and guidelines of the Research and Ethical Clearance committee (RECC) of Addis Ababa University which is called CNS-IRB. Woreda (district) health, kebele (subdistrict) administrators, and health extension workers have briefed the objective of the study. Households gave consent for their child's involvement in dietary data collection and dental and skeletal fluorosis assessment. The study participants (caregivers and children) were informed about the objective of the study.

## Results

### Subjects

There were 127 children who completed this study, which is a response rate of 94% among 135 children initially recruited. Socio-demographic characteristics of the households and children enrolled in this study are shown in Table 1. The mean age (SD) of school children was 7.8 (1.9) years. About one-third (34.6%) of school-age children had not attended formal school education. The main source of income for all households included in this study was farming. Most (92.5%) of the households were Muslim by religion. For the majority (67.7%) of households, motor pump groundwater was the source of drinking water.

**Table 1:** Socio-demographic characteristics of school-age children (*n* = 127) aged 6–13 years from selected one kebeles, Halaba zone, SNNPR Ethiopia, 2019.

**Variables**	**Frequency (%)**
**Children's age (y)**	
6–8	67 (52.8)
9–13	60 (47.2)
**Children's sex**	
Male	68 (53.5)
Female	59 (46.5)
**Educational status of children**	
In school	83 (65.4)
Not in school	44 (34.6)
**Household occupation**	
Farming	127 (100)
**Household Religion**	
Orthodox Christian	2 (1.6)
Muslim	118 (92.9)
Protestant Christian	7 (5.5)
**Agricultural land size**	
< /= ½ Hectare	108 (85)
> ½ Hectare	19 (15)
**Sources of drinking water**	
Motor pump groundwater	86 (67.7)
Spring water	36 (28.3)
Tap water	5 (3.9)

### Biochemical Results

The F level in drinking water from different sources averaged by taking % usage into consideration 5.09 mg/L. The content of F in all three community drinking water supply sources exceeded the WHO limit (1.5 mg/L) (Table 2).

**Table 2:** Fluoride (F) levels of water collected from different sources from Halaba SNNPR, Ethiopia, 2019.

**Water type**	**F level mg/L**	**Use by Households**	**Average over community**
		**(%)**	**(mg/L)**
Tap water	4.73	67.7	5.09
Motor pump groundwater	6.21	28.3	
Spring water	3.33	3.9	

The F content of selected food items varied between 0.83 and 13.61 mg/kg as shown in Table 3. Food items that were prepared from maize and millet, the staple foods of the community, had the highest concentration of F. In contrast, vegetable sources such as cabbage and potato had low concentrations of F. For all food items, % recovery was varied between 90.4 and 107.9%, which falls in an acceptable range.

**Table 3:** Fluoride levels of foods and beverages collected from Halaba zone SNNPR Ethiopia, 2019.

**Sample Type**	**F (mg/kg)**	**Recovery (%)**
Injera (unknown ingredients)	10.61	100.64
Unleavened bread (unknown ingredients)	13.61	97.25
Unleavened bread, maize	12.51	102.48
Injera (maize, millet (1:1))	8.28	102.54
Unleavened bread, millet	4.89	93.70
Injera [sorghum, maize, millet (1:1:1)]	4.95	95.36
Bread (maize)	10.49	99.02
Injera (millet)	7.84	105.68
Boiled potato and carrot	1.6	92.21
Rice with carrot	3.18	103.64
Shiro stew without tomato	3.87	103.60
Boiled cabbage	0.83	103.18
Banana	5.26	107.89
Taro, *Colocasia antiquorum*: boiled	4.61	96.19
Boiled beetroot	3.2	102.68
Cabbage and potato stew	2.42	95.92
Coffee (prepared from coffee leaf)	3.5	104.45

#### Dietary Diversity and Dietary Intakes

**Figure 1:**
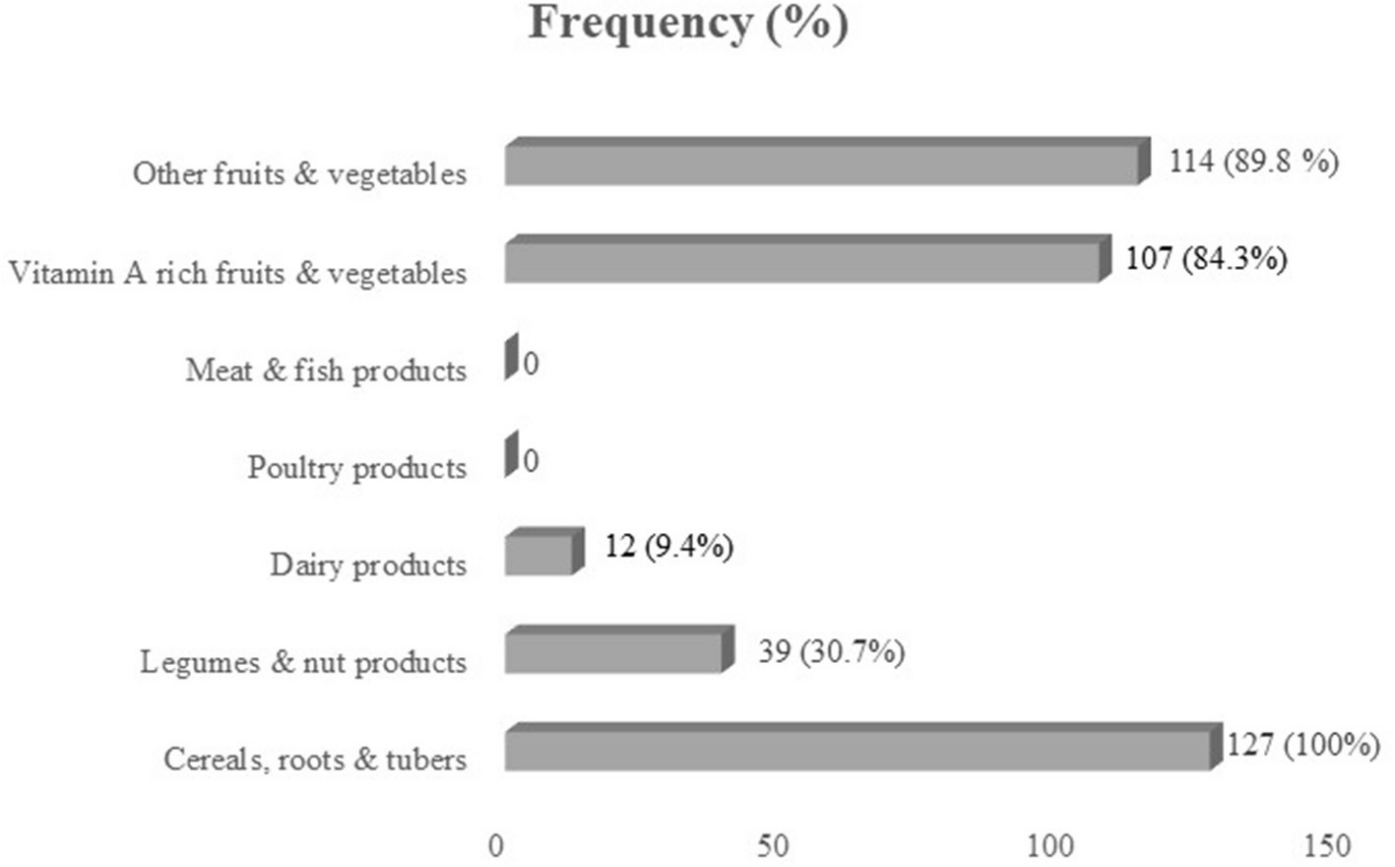
Consumption (%) of seven food groups by school-age children in Halaba zone, SNNPR Ethiopia.

Out of seven food groups, the mean diet diversity score was 3.13. The diet was predominantly cereal-based, consumed by all children (100%). Legumes and nut products were consumed by 30.7% of children, whereas 9.4% of children consumed dairy products. Poultry products, meat, and fish products were not consumed at all by children during the assessment. Vitamin A-rich fruits and vegetables were consumed by 84.3% of children, whereas 89.8% of children consumed other fruits and vegetables (Figure 1).

#### Calcium Intakes

The calcium intake of these school children averaged 520 mg/day over both age groups when assessed by 24-h recall, and 452 mg/day when assessed by the FFQ (Table 4). Data from these two dietary assessment methods were highly correlated (*r* = 0.728, *P* < 0.001). The FFQ which asked only about the good sources of calcium underestimated intake by only 70 mg. Intakes of the children were well-below the RDA for each age group [23].

**Table 4:** Mean (SD) dietary calcium (Ca) and total fluoride (F) intakes of school children 6–13 years, mean (SD) in comparison with dietary standards, Halaba Zone selected kebele, SNNPR, Ethiopia (2019).

**Intake**	**Children**	**Children**	**All children**
**mg/day**	**6–8 y**	**9–13 y**	**6–13 y**
	*N* = 67	*N* = 60	*N* = 127
**Ca**			
By 24-h recall	544 (203)	493 (205)	520
By FFQ	449 (148)	457 (143)	452
RDA	1,000	1,300	
UL	2,500	3,000	
**Total F**			
By 24-hour recall	10.3 (3.4)	10.9 (3.7)	10.6
By FFQ	9.0 (0.97)	9.0 (0.93)	9.0
AI	1.0	2.0	
UL	2.2	10	

*RDA, recommended dietary allowance; AI, Adequate Intake; UL, Upper Tolerable Intake Level. Recommended values for calcium [23] and for F [1] from the Institute of Medicine*.

#### Fluoride Intakes

The mean total F intake, from food and water, was ~10 mg/day by 24-h recall, and 9 mg by FFQ. These intakes were well-above the UL of F for the younger school children and close to the UL for the older children [1]. The intakes of F are summarized in Table 4.

#### Dental Fluorosis

**Figure 2:**
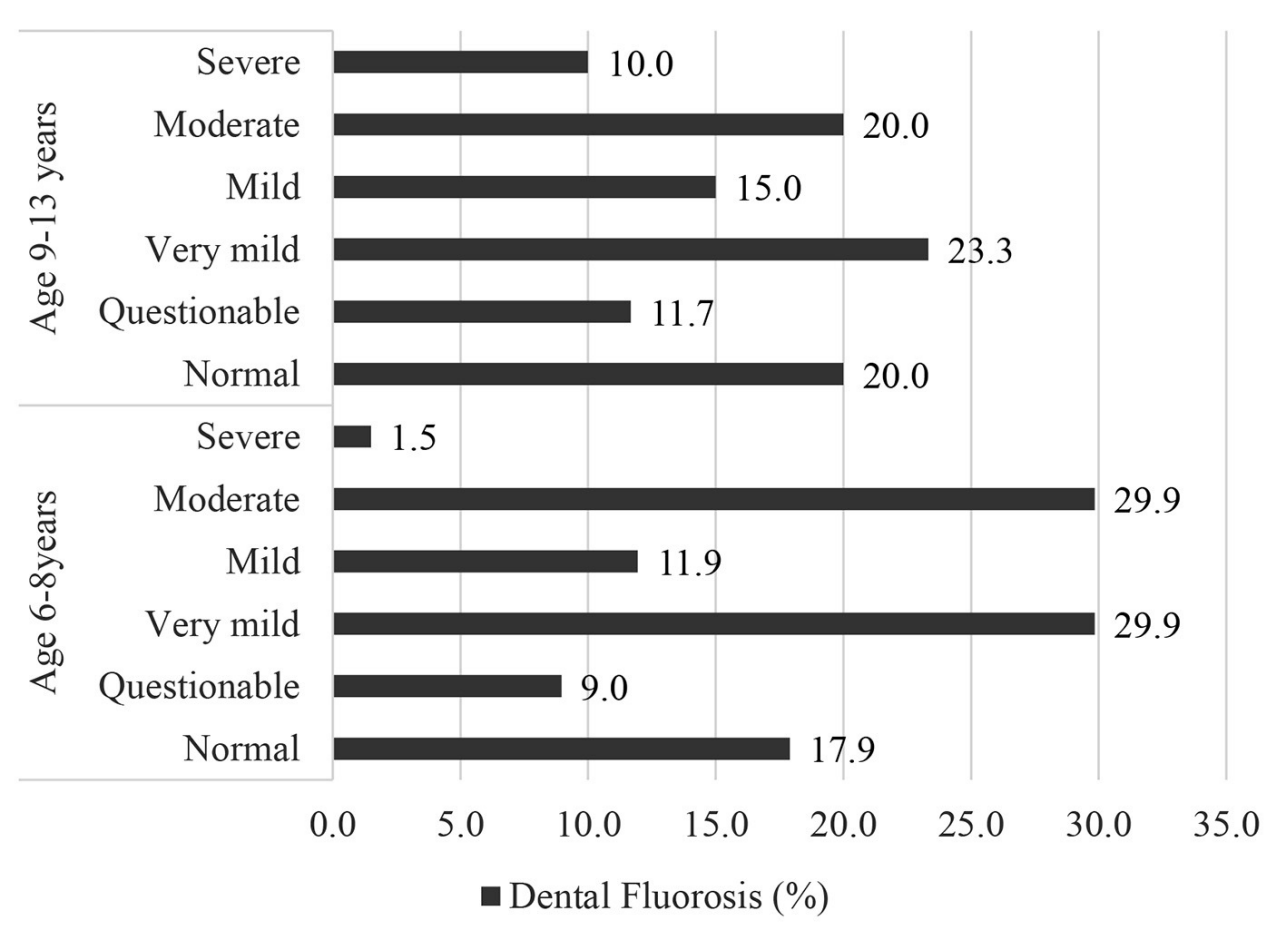
Prevalence (%) of dental fluorosis among school children (*N* = 127) in Halaba, SNNPR Ethiopia, 2019.

The overall prevalence of dental fluorosis (from very mild to severe symptoms) was 73.1% for the younger children (6–8 years) and 68.3% for the older children (9–13 years) (Figure 2), and prevalence of severe dental fluorosis was 1.5 and 10.0%, respectively. This shows that in this community, more older children were experiencing dental problems due to the high F in water and foods, than younger children.

#### Skeletal Fluorosis

**Figure 3:**
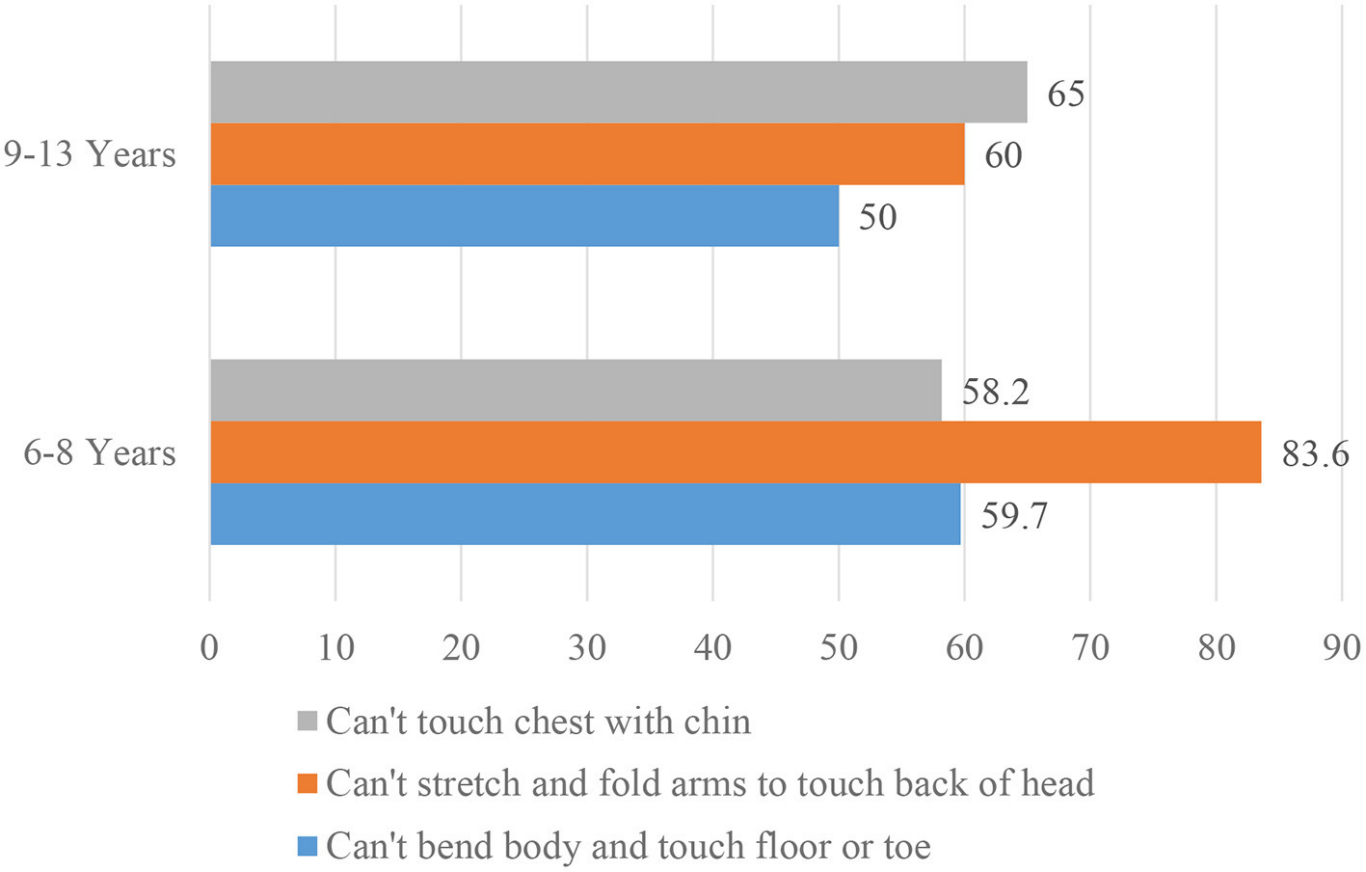
Prevalence (%) of skeletal fluorosis (SK) among school children (*N* = 127) in Halaba, SNNPR Ethiopia, 2019. The tests for SK are: SF Test-1 is “can't bend body touch floor or toe”; SF Test-2 is “can't stretch and fold arms to touchback of the head”; SF Test-3 “can't touch the chest with the chin”).

The prevalence of children having symptoms of skeletal fluorosis ranged between 55.1 and 72.4%. More than half of the children (55.1%) were unable to bend the body and touch the floor or toe. Around two-third (61.4%) of the children were unable to touch their chest with their chin, and over two-third (72.4%) of children were unable to stretch and fold arms to touch the back of the head (Figure 3). There was no apparent age difference in ability to do these exercises.

### Associations Between Calcium and Fluoride Intakes and Fluorosis

As shown in Table 5, total F intake of children was significantly positively associated [AOR (95% CI) 3.486 (1.229, 9.885)] with the presence of dental fluorosis. Dietary Ca intake of children was significantly negatively associated [AOR (95% CI) 0.170 (0.065, 0.444)] with dental fluorosis level. Thus, while high F intake was related to a high prevalence of dental fluorosis, low Ca intake by children was also related to high levels of dental fluorosis. However, dietary Ca intake was not associated with total F intake.

**Table 5:** Predictors of dental fluorosis (DF) among school children using calcium (Ca) and fluoride (F) intakes from 24-h dietary data: logistic regression model, Halaba zone, SNNPR Ethiopia, 2019.

**Variable^a^ (*n* = 127)**	**Dental fluorosis^b^**	
	**COR (95%CI)**	**AOR (95%CI)**
Dietary F (>10.57mg/day)	5.402 (2.054, 14.205)^**^	3.486 (1.229, 9.885)^*^
Dietary Ca (>520mg/day)	0.138 (0.056, 0.338)^**^	0.170 (0.065, 0.444)^**^
Age (9-13 years)	0.793 (0.368, 1.706)	0.510 (0.203, 1.283)
Sex (Female)	0.883 (0.410, 1.901)	0.689 (0.278, 1.706)

a *The reference groups for each variable are: Dietary F ≤ 10.57 mg/day, Dietary Ca ≤ 520 mg/day, Age (6–8 y) and Sex (male), respectively*.

b *The six symptoms of dental fluorosis were categorized into categorical variable (present=1 or absent =0) regression analysis. The first group was DF absent (normal and questionable symptoms) and the second group was any DF present (very mild, mild, moderate, and severe symptoms)*.

* *P < 0.05*,

** *P < 0.01*.

Higher than average total F intake of children significantly increased the odds of developing skeletal fluorosis symptoms as measured as inability to stretch and fold arms to touch back of head (SF-test 2) both as unadjusted and adjusted OR values (*P* < 0.05); however, no significant effect were noted for the other two tests (Table 6). Higher than average Ca intake was negatively associated (at *P* < 0.05) with each test without adjustment, but only one test showed a significant reduction in the odds of developing skeletal fluorosis which was SF test-1, i.e., unable to bend body to touch the toes or floor. Effects of age on performing SF tests was highly variable. Older children had greater odds of developing skeletal fluorosis measured as SF-test 2, even when adjusted. Sex of the child was not a factor.

**Table 6:** Predictors of fluorosis symptoms (Skeletal fluorosis) among school children from 24-h dietary data: logistic regression model, Halaba zone, SNNPR Ethiopia, 2019.

**Variable^A^ (*n*= 127)**	**SF Test-1^a^**		**SF Test-2^b^**		**SF Test-3^c^**	
	**COR (95% CI)**	**AOR (95% CI)**	**COR (95% CI)**	**AOR (95% CI)**	**COR (95% CI)**	**AOR (95% CI)**
Dietary F (>10.57 mg/day)	2.116 (1.030, 4.348)^*^	1.577 (0.725, 3.431)	2.952 (1.323, 6.586) ^**^	2.454 (1.006, 5.986)^*^	1.714 (0.829, 3.545)	1.397 (0.638, 3.061)
Dietary Ca (>520 mg/day)	0.375 (0.181, 0.775)^**^	0.453 (0.208, 0.984) ^*^	0.420 (0.185, 0.956)^*^	0.645 (0.257, 1.619)	0.455 (0.218, 0.952)^*^	0.476 (0.215, 1.055)
Age (9–13 y)	1.481 (0.733, 2.993)	1.388 (0.658, 2.926)	3.394 (1.484, 7.763) ^**^	3.266 (1.370, 7.786) ^**^	0.750 (0.365, 1.539)	0.631 (0.295, 1.351)
Sex (Female)	1.215 (0.602, 2.450)	1.280 (0.609, 2.692)	0.960 (0.439, 2.096)	1.129 (0.481, 2.651)	0.903 (0.441, 1.850)	0.812 (0.384, 1.719)

* *P < 0.05*,

** *P < 0.01*.

*SF, skeletal fluorosis; Ca, calcium; F, fluoride*.

a *SF Test-1 is “can't bend body touch floor or toe”*.

b *SF Test-2 is “can't stretch and fold arms to touch back of the head”*.

c *SF Test-3 “can't touch the chest with the chin”*.

A *The reference groups for each variable are: Dietary F ≤ 10.57 mg/day, Dietary Ca ≤ 520 mg/day, Age (6–8 y) and Sex (male), respectively*.

## Discussion

In this study, a high prevalence of dental fluorosis among school-age children was shown for this community as was the presence of skeletal fluorosis, as reported earlier [13]. For the first time we report individual dietary F intakes of the school children, which reached UL values, and individual dietary Ca intakes which were well-below the recommended intake. Our findings show that excess dietary F intake and low consumption of dietary Ca increased the odds of having dental and skeletal fluorosis in school-age children living in the Ethiopian Rift Valley.

The mean F intake by children was above the upper tolerable-intake level of the Institute of Medicine [1] for young children age 6–8 year and close to the UL for older children. Water is the major source of excess F intake in that community; however, food and other beverages also had a significant contribution to excess F intake by the children. This finding supports previous studies in the Ethiopian Rift Valley [14, 23] and confirms that high F remains a problem in this area.

Dental fluorosis is a dose-response effect caused by excess F ingestion during the pre-eruptive development of teeth [1]. According to the results obtained in this study, the overall prevalence of dental fluorosis (very mild to severe forms) in school-age children was very high at 73%, wherein 25% of children showed moderate dental fluorosis (all surfaces affected, with some brown spots and marked wear on surfaces subject to attrition) and 5.5% were affected by severe dental fluorosis (widespread brown stains and pitting). Previously it was reported that in Halaba 17% of school-age children had moderate dental fluorosis and 3% were severe [13], and differences may reflect a trend toward increased incidence or may be the result of a difference in assessment by dentists.

Dietary F ingestion over 10.57 mg/day, in our adjusted model, increased the odds of developing dental fluorosis by ~350%. After adjusting for F intake, a dietary Ca intake over >520 mg/day reduced the odds of dental fluorosis by 83% in the children. According to Cerklewski [24], Ca forms insoluble complexes with F which then decreases F absorption and its subsequent uptake into bone and teeth. It was shown over three decades ago that when F is ingested together with Ca-rich foods, the bioavailability of F decreases by 40% [25]. An animal study confirmed this relationship, finding increased fecal F when dietary Ca was administered to rats subjected to a high F intake [13]. Dietary Ca intake of the children in our study community was well-below recommendations, a finding that adds to the growing body of research showing dietary Ca is a nutrient of concern in Ethiopia [26, 27]. While milk was not a source of Ca in our research site, a study conducted in the Ziway-Shala basin of Ethiopia found that milk consumption in 1,000 people (average age 17 year) was inversely associated with dental fluorosis severity [9], in agreement with our findings. That study, however, did not measure skeletal fluorosis.

The results of skeletal fluorosis which was done using physical exercise tests showed that many children could not bend the body to touch the floor (SF Test-1), could not fold arms to touch the back of the head (SF Test-2), and/or or could not touch the chest with chin (SF Test-3). These results agree with an earlier study conducted in Ethiopia, which found that linear relationships between the development of skeletal fluorosis and F concentration of drinking water [28]. No report, however, has previously shown that dietary Ca was a factor in skeletal fluorosis. Kebede et al. [13] saw differences in prevalence of skeletal fluorosis symptoms of school-age children in Ethiopia and hypothesized that dietary Ca was a factor in differences between communities having similar water F levels. Our data showed a modest but significant effect of increased dietary Ca on improving the odds of children to be able to bend the body to touch floor or toe by 55%. We also showed that high F intake reduced the odds of being able to stretch and fold arms to touch back of the head by close to 250%. Data from young children was highly variable yet it does provide support for the findings of epidemiological research that an intake of at least 10 mg/day for 10 or more years is needed to produce clinical signs of the milder forms of skeletal fluorosis [1].

Our study has several limitations. First, the cross-sectional nature does not allow inferences to be made on causality and rather can only indicate associations of dietary intake with chronic effects (fluorosis). Further, while dental fluorosis can be detected by qualified dental professional, the symptoms for skeletal fluorosis assessment that we used were indirect indicators, and radiographs are required to diagnose this condition. Further, we did not measure the intra-examiner reliability. Dietary data by 24-h recall and collection of some food and water samples were done 1 year later than assessment of fluorosis signs and symptoms due to scheduling difficulties. Furthermore, some food composition data for F in foods that were not directly measured were taken from an American database which may vary from the Ethiopian diet.

## Conclusions

The presence of dental and skeletal fluorosis in school-age children indicated a major health concern in the community, one that starts in childhood and continues throughout life. The dietary F intake was at or above the upper tolerable intake level of 0.1 mg/kg/body weight recommended by the Institute of Medicine [1]. Water was the major source of F, followed by food and beverage sources. The water F level (averaging 5.1 mg/L) consumed by the community exceeded the WHO limit (1.5 mg/L) for drinking water. The dietary Ca intake of children was well-below the recommended intake levels. A simple FFQ may be used to estimate intakes of both F and Ca. Our data support the hypothesis that in the presence of excess F intake, a higher-than-average consumption of dietary Ca may mitigate the severity of dental fluorosis and skeletal fluorosis symptoms in children. This does not mean that water de-fluoridation efforts should be abandoned, but efforts for the latter have not always been successful 29). Hence, an educational intervention to improve Ca status such as promoting greater consumption of Ca-rich foods such as Ethiopian kale, millet, and Enset is warranted in areas experiencing fluorosis where Ca intakes are low.

## Data Availability Statement

The raw data supporting the conclusions of this article will be made available by the authors, without undue reservation.

## Ethics Statement

The studies involving human participants were reviewed and approved by Addis Ababa University Ethical Approval Committee. Written informed consent to participate in this study was provided by the participants' legal guardian/next of kin.

## Author Contributions

NT, DM, KB, MT, and SW participated in the conception study design. NT, DM, MW, and AY participated in data collection. Analysis of data was by NT. The first draft of the work was by NT. All authors revised the manuscript critically for important intellectual content and reviewed the manuscript and approved the final version to be published.

## Funding

The financial support was covered by Addis Ababa University, Ethiopian Public Health Institute, and Hawassa University.

## Conflict of Interest

The authors declare that the research was conducted in the absence of any commercial or financial relationships that could be construed as a potential conflict of interest.

## Publisher's Note

All claims expressed in this article are solely those of the authors and do not necessarily represent those of their affiliated organizations, or those of the publisher, the editors and the reviewers. Any product that may be evaluated in this article, or claim that may be made by its manufacturer, is not guaranteed or endorsed by the publisher.

## Abbreviations

AI: Adequate Intake
AOR: Adjusted Odd Ratio
Ca: Calcium
CI: Confidence interval
EDHS: Ethiopia Demographic and Health Survey
F: Fluoride
FFQ: Food frequency questionnaire
IOM: Institute of Medicine
OR: Odds Ratio
SNNPR: Southern Nation Nationality and Peoples Region
UL: Upper Level (or upper limit)
WHO: World Health Organization.
